# Supplementary figures and images for: Systematic transformation of urban cold chain networks: From cross-regional dependencies to sustainable local excellence
Source: PLoS One. 2025 Nov 25;20(11):e0336993. doi: 10.1371/journal.pone.0336993 (PMC12646484; doi:10.1371/journal.pone.0336993)

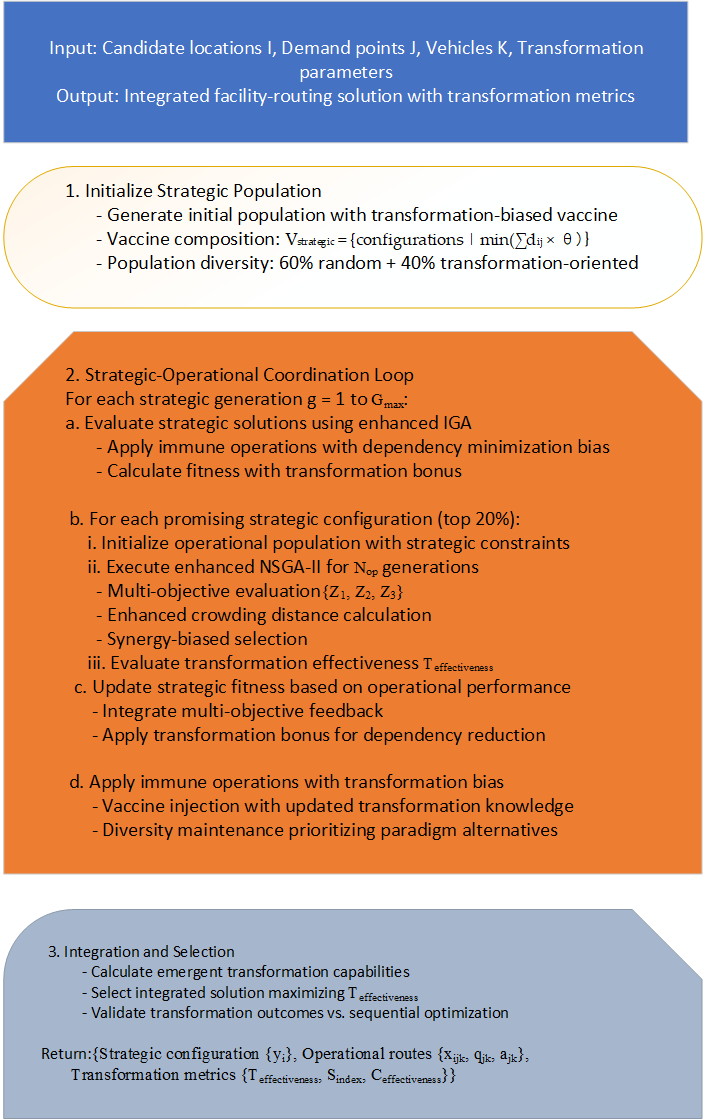

Supplement: S1 File — This single file contains all supplementary materials, organized into the following sections: Theoretical Framework and Mathematical Proofs (S1), Parameter Calibration and Enterprise Validation (S2), Detailed Validation Protocol and Metrics S3), Algorithm Details (S4), Ablation Study: Detailed Results and Configuration S5), Store Classification System Validation (S6), Case Study Background: Candidate Distribution Centers (S7), Complete Statistical Analysis and Validation Results S8), Comprehensive Algorithm Performance Analysis S9), Comprehensive Stochastic Robustness Analysis (S10), Supplementary Analysis for Boundary Conditions (S11), Detailed Cross-City Cluster Applicability Analysis (S12), Supplementary Robustness Analysis for Generalizability S13). (ZIP) [file pone.0336993.s001.zip › Fig/Figure S1.tif]

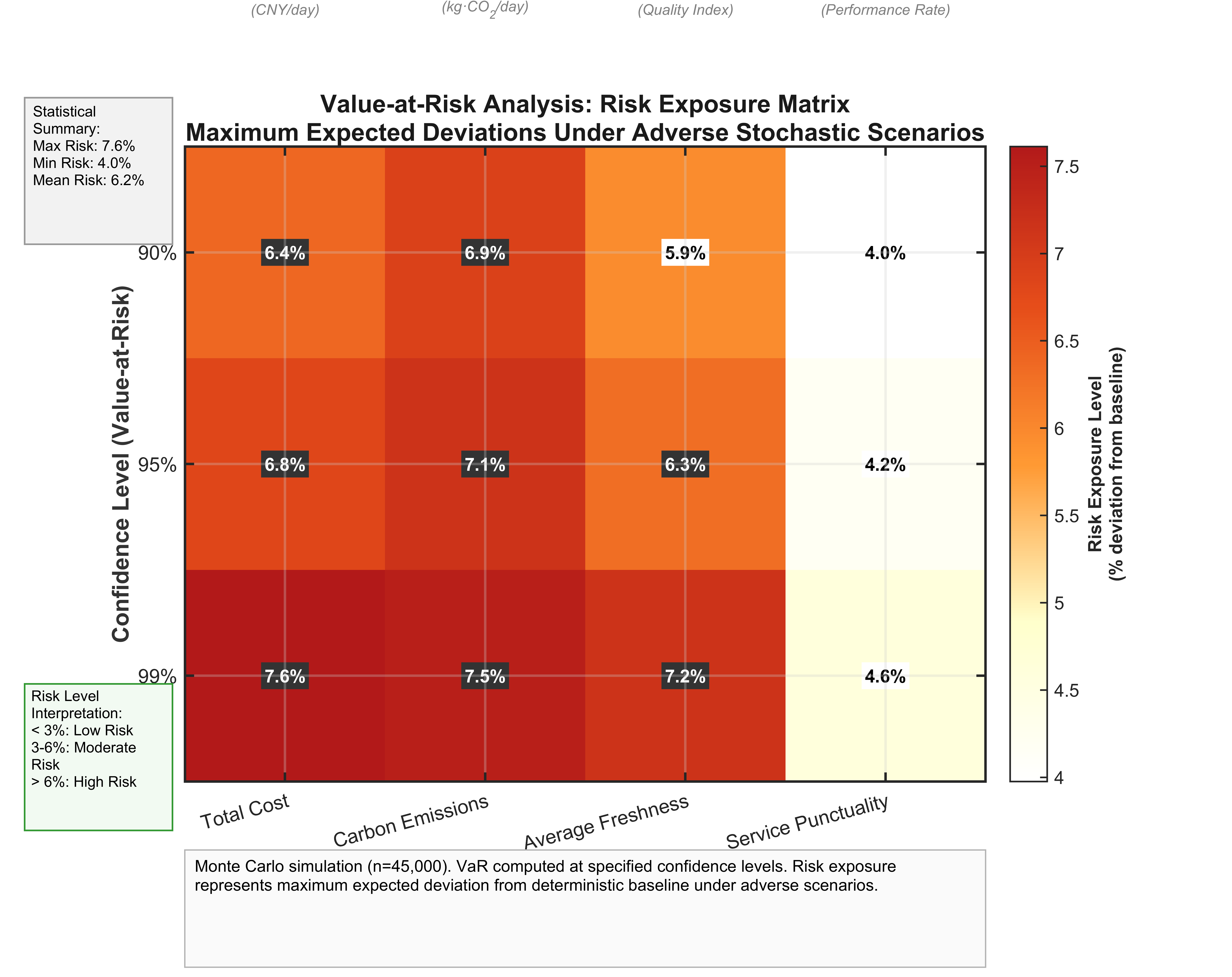

Supplement: S1 File — This single file contains all supplementary materials, organized into the following sections: Theoretical Framework and Mathematical Proofs (S1), Parameter Calibration and Enterprise Validation (S2), Detailed Validation Protocol and Metrics S3), Algorithm Details (S4), Ablation Study: Detailed Results and Configuration S5), Store Classification System Validation (S6), Case Study Background: Candidate Distribution Centers (S7), Complete Statistical Analysis and Validation Results S8), Comprehensive Algorithm Performance Analysis S9), Comprehensive Stochastic Robustness Analysis (S10), Supplementary Analysis for Boundary Conditions (S11), Detailed Cross-City Cluster Applicability Analysis (S12), Supplementary Robustness Analysis for Generalizability S13). (ZIP) [file pone.0336993.s001.zip › Fig/Figure S2.tif]

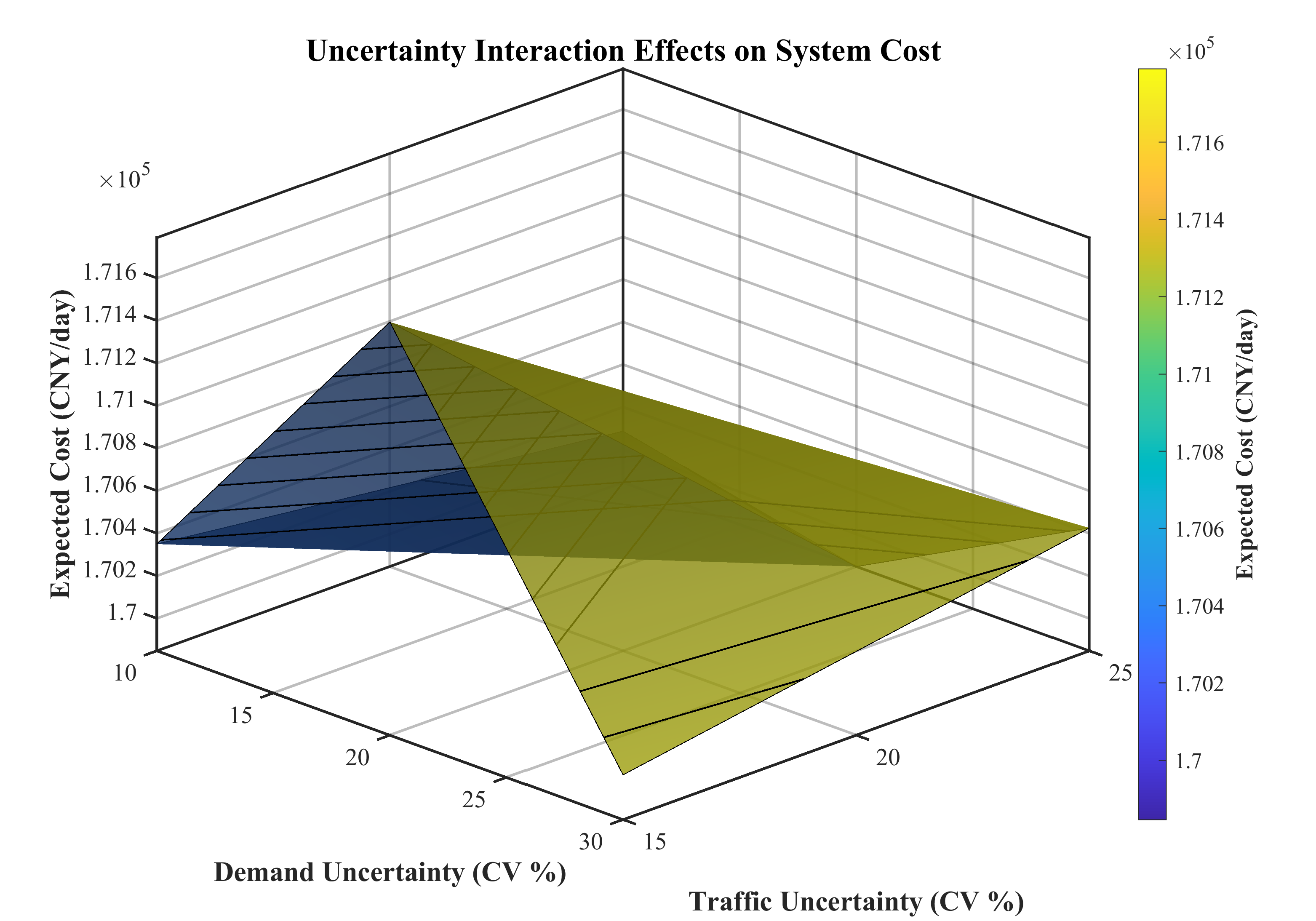

Supplement: S1 File — This single file contains all supplementary materials, organized into the following sections: Theoretical Framework and Mathematical Proofs (S1), Parameter Calibration and Enterprise Validation (S2), Detailed Validation Protocol and Metrics S3), Algorithm Details (S4), Ablation Study: Detailed Results and Configuration S5), Store Classification System Validation (S6), Case Study Background: Candidate Distribution Centers (S7), Complete Statistical Analysis and Validation Results S8), Comprehensive Algorithm Performance Analysis S9), Comprehensive Stochastic Robustness Analysis (S10), Supplementary Analysis for Boundary Conditions (S11), Detailed Cross-City Cluster Applicability Analysis (S12), Supplementary Robustness Analysis for Generalizability S13). (ZIP) [file pone.0336993.s001.zip › Fig/Figure S3.tif]
