# Supplementary material for: Systematic transformation of urban cold chain networks: From cross-regional dependencies to sustainable local excellence
Source: PLoS One. 2025 Nov 25;20(11):e0336993. doi: 10.1371/journal.pone.0336993 (PMC12646484; doi:10.1371/journal.pone.0336993)
Supplement: Attachment — Submitted filename: Response to Reviewers.docx [file pone.0336993.s003.docx]

**Manuscript ID: PONE-D-25-41349
Title: Systematic Transformation of Urban Cold Chain Networks: From Cross-Regional Dependencies to Sustainable Local Excellence**

**Dear Reviewers,**

**Thank you for the opportunity to revise and resubmit our manuscript. We are grateful to you and the reviewers for the insightful and constructive feedback. We found the comments to be exceptionally helpful, and they have guided us in a substantial revision that we believe has significantly strengthened the paper's scientific contribution, clarity, and impact.**

**In response to the feedback, we have undertaken a comprehensive effort that goes beyond minor corrections. This includes a complete restructuring of the manuscript into four core chapters (1-Theory, 2-Methodology, 3-Empirical Validation, 4-Conclusions) for improved logical flow. The other major enhancements include:**

1. **A completely redesigned four-tier comparative analysis to transparently disentangle the effects of systemic restructuring versus our algorithmic enhancements.**
2. **The introduction of extensive new analyses, including a full ablation study, a large-scale stochastic robustness analysis, and a novel boundary analysis to define practical performance limits.**
3. **A significant rewriting of the theoretical framework to more rigorously establish its originality and quantitative contributions.**
4. **A complete redesign of all key figures to meet the highest standards of scientific visualization.**

**We are confident that these extensive revisions have fully addressed all concerns raised. Below, we provide a detailed, point-by-point response.**

**Responses to Reviewer #1**

**Comment 1-1: On the fairness of the performance comparison.**

**Response:
We thank the reviewer for this critical and insightful comment. We completely agree that attributing the entire performance gain to our algorithm would be an overstatement. To provide a more nuanced and transparent analysis, we have completely redesigned our empirical validation framework.**

**Action Taken: We have introduced a comprehensive four-tier comparative analysis (now in Section 3.2). This new framework, visualized in Figure 8 and detailed in Table 7, allows us to rigorously disentangle the different sources of improvement. We now clearly show that while system restructuring is the primary driver (56.2% of the gain), our specific algorithmic enhancements contribute a statistically significant but realistic 13.7% of the total improvement over other state-of-the-art algorithms.**

**Comment 1-2: On the originality and necessity of the theoretical contributions.**

**Response:
We appreciate the opportunity to clarify the theoretical originality of our work.**

**Action Taken: In the revised Section 1.1 (Systematic Transformation Theory), we now formally introduce and define our three novel, quantifiable mathematical constructs (**$T_{effectiveness}$**,** $S_{index}$**,**$D_{coefficient}$**). The updated Table 1 provides a direct comparison to established qualitative theories, demonstrating how our work extends them with crucial mathematical formalization.**

**Comment 1-3: On the impact of strong assumptions and the need for robustness analysis.**

**Response:
This is an excellent point. To enhance the practical relevance of our model, we have conducted extensive new analyses.**

**Action Taken:**

1. **We have added Section 1.5: Model Assumptions, Limitations, and Performance Evaluation, which quantifies the impact of relaxing key assumptions.**
2. **More significantly, we have introduced a large-scale stochastic robustness analysis (now Section 3.6). Based on 45,000 Monte Carlo simulations (Table 12 and Figure 14), we demonstrate that our framework maintains remarkable robustness, retaining over 94% of its benefits under realistic operational volatility.**

**Comment 1-4: On the need for an ablation study.**

**Response:
We thank the reviewer for this valuable suggestion.**

**Action Taken: We have introduced a new Section 1.6: Ablation Study of Algorithmic Contributions. This study (Figure 3) systematically isolates and quantifies the individual contributions of each algorithmic component, providing rigorous validation for our methodological design.**

**Comment 1-5 & 1-6: On streamlining the text and adding literature.**

**Response:
We have followed the reviewer's suggestions. The manuscript has been significantly streamlined into a four-chapter structure, with extensive details moved to the Appendix. All three recommended articles have been incorporated.**

**Responses to Reviewer #2**

**We thank Reviewer #2 for the positive assessment and the detailed, constructive feedback, which has been invaluable in elevating the manuscript. We have systematically addressed all points raised.**

- **On Theoretical Positioning, Assumptions, and Limitations (R2-1, R2-2, R2-3): We have addressed these points through the revised Section 1.1 and Table 1 (clarifying theoretical advancements), the new Section 1.5 (discussing model limitations and assumption impacts), and the comprehensive stochastic analysis in Section 3.6.**
- **On Methodological Rigor (R2-4, R2-5, R2-6, R2-7): We have provided enhanced details on parameter calibration (Section 1.4), a full computational complexity analysis (Sections 2.7 & 3.4, Figure 11), a more credible performance validation via the four-tier analysis (Section 3.2), and a rigorous validation of our store classification system (Section 3.1, Table 5). To address the core concern of R2-6, we have also added a novel Boundary Analysis (Section 3.7, Figure 15) to explore the full spectrum of achievable performance.**
- **On Manuscript Presentation (R2-8, R2-9, R2-10): The manuscript has been completely restructured and condensed. All key figures have been redesigned. The abstract has been rewritten to be more accessible, highlighting the key practical outcome: a 44.1% reduction in both cost and carbon emissions.**
- **On Broader Impact and Practical Implications (R2-11, R2-12, R2-13): We have added three substantial new sections to address these crucial points:**
  - **Section 3.8 (Cross-Urban Agglomeration Transformation Applicability) analyzes the framework's performance across ten representative urban agglomerations.**
  - **Section 4.7 (Policy Implementation Framework) provides concrete policy recommendations.**
  - **Section 4.4 (Implementation Challenges, Risk Management, and Strategic Pathways) has been significantly expanded to discuss barriers such as upfront investment (CNY 47.15 million) and organizational resistance.**

**We believe that these extensive revisions, guided by the reviewers' expert feedback, have transformed the manuscript into a much stronger, more rigorous, and more impactful piece of research. We are grateful for the opportunity to improve our work and hope that you will now find it suitable for publication in *PLOS ONE*.**

**Sincerely,**

**Yuhong Chen (on behalf of all authors)
School of Economics and Management
Inner Mongolia University of Technology**
