# Supplementary material for: Systematic transformation of urban cold chain networks: From cross-regional dependencies to sustainable local excellence
Source: PLoS One. 2025 Nov 25;20(11):e0336993. doi: 10.1371/journal.pone.0336993 (PMC12646484; doi:10.1371/journal.pone.0336993)
Supplement: Attachment — Submitted filename: Response_to_Reviewers_auresp_2.docx [file pone.0336993.s004.docx]

**Manuscript ID: PONE-D-25-41349
Title: Systematic Transformation of Urban Cold Chain Networks: From Cross-Regional Dependencies to Sustainable Local Excellence**

**Dear Reviewers,**

**We sincerely thank the Academic Editor and both expert reviewers for their invaluable feedback. In response to the second round of reviews, we have undertaken a comprehensive and substantial revision that we believe has significantly strengthened the manuscript.**

**Key enhancements in this revision include:**

- **A sharper articulation of our theoretical framework's originality and its advancement beyond existing qualitative paradigms.**
- **An in-depth methodological explication of the emergent capabilities arising from our hierarchical architecture.**
- **New analyses and discussions to bolster the claims of generalizability and scalability.**
- **A thorough contextualization of our carbon pricing parameter with global benchmarks and a new policy framework to address implementation equity.**
- **A significant streamlining of the entire manuscript for clarity and impact, with technical details strategically moved to the supplementary materials.**

**We are confident that these revisions fully address all the reviewers' concerns. Below is our detailed point-by-point response.**

**Response to Reviewer #1**

**We note that no new comments were provided in the second round of review. We once again thank Reviewer #1 for their insightful feedback in the initial review, which was instrumental in shaping the current, much-improved version of our manuscript.**

**Response to Reviewer #2**

**We are deeply grateful to the reviewer for their positive assessment and for providing such insightful and constructive suggestions in the second round. These comments have guided us in significantly elevating the quality, clarity, and impact of our manuscript.**

**Comment #1: The distinction between "Systematic Transformation Theory" and existing theories (e.g., Geels' Multi-Level Perspective) needs sharper articulation. Explicitly contrast how your quantitative framework advances beyond qualitative paradigms.**

**Response: We thank the reviewer for this crucial suggestion. We have substantially strengthened our theoretical positioning in two key ways:**

1. **In the Introduction (Lines 55-69), we have inserted a new section that explicitly contrasts our theory with existing qualitative frameworks. We now clearly state that while frameworks like Geels' MLP provide a powerful qualitative lens, they inherently lack the mathematical mechanisms for quantifying transformation effectiveness or predicting successful paradigm shifts.**
2. **Following Table 1 (Lines 120-139), we have added a detailed exposition that systematically illustrates how our three novel metrics (**$T_{effectiveness}$**,**$S_{index}$**, and** $D_{coefficient}$**) enable a fundamental shift from qualitative description to quantitative prediction, measurement, and validation.**

**Comment #2: The ablation study could better highlight why the hybrid algorithm outperforms standalone algorithms. Provide a deeper discussion on the "emergent capabilities" and their theoretical implications.**

**Response: We agree this is a critical point. To address it, we have replaced the original Section 2.4 with a new, in-depth section (Section 2.4: Methodological Insight: Emergent Capabilities from Hierarchical Coordination, Lines 387-412). This new section provides the deep theoretical explanation requested, detailing the bidirectional information feedback loops, the mechanisms of "Top-Down Enablement" and "Bottom-Up Feedback," and how the Transformation Effectiveness Coefficient (**$T_{effectiveness}$**= 1.34) serves as the empirical quantification of the emergent benefit arising from fundamental architectural advantages.**

**Comment #3: Clarify how the 35-store sample represents broader urban agglomerations and include sensitivity analysis on scalability (e.g., N > 500).**

**Response: We thank the reviewer for this important point on external validity. We have provided a two-pronged response in the main text:**

1. **Sample Representativeness (Section 3.1, Lines 510-519): We have added a new paragraph clarifying that our 35-store sample is a typologically representative sample, scientifically validated via clustering analysis (Table 5) to cover three distinct urban store formats (Type A, B, C). We also highlight that our cross-urban validation (Section 3.8) confirms the framework's broad applicability.**
2. **Scalability Projection (Section 3.4, Lines 628-638): We have added a new subsection providing concrete runtime estimations for N > 500. These projections are rigorously derived from the empirically validated computational complexity of** $O\left(n^{1.91}\right)$**. The detailed analysis for this is provided in Supporting Information S9.4.**

**Comment #4: Address how the framework adapts to low-probability, high-impact events (e.g., pandemic-scale demand shocks).**

**Response: We appreciate this forward-thinking observation. We have added a new subsection at the beginning of our future research section (Section 4.5.1: Toward a Dynamic and Stochastic Transformation Theory for Resilience, Lines 823-838). This section acknowledges this as a future research area, theoretically posits the framework’s inherent resilience (supported by data from Table 6), clarifies the need for different methodologies (e.g., agent-based modeling), and frames "Resilient Transformation" as a high-priority research direction.**

**Comment #5: The carbon pricing recommendation lacks contextualization with global benchmarks and a discussion of equity issues.**

**Response: This is a critical point, and we have undertaken a major revision:**

1. **Benchmarking (Note for Table 3, Lines 198-211): We have added a detailed clarification explaining that our**$\theta$**parameter is a comprehensive economic parameter. We isolate its direct carbon price component (~60 CNY/tonne CO₂) and demonstrate its consistency with the China National ETS, EU ETS, and California Cap-and-Trade program.**
2. **Equity Issues (Section 4.7, Lines 867-892): We have substantially restructured this section to propose an evidence-based policy framework with three integrated mechanisms for equitable implementation.**

**Comment #6: Ensure all figures are legible.**

**Response: We thank the reviewer for this reminder. All figures have been regenerated at ≥300 DPI in TIFF/EPS format. We have paid careful attention to all graphical elements, including font sizes, line weights, and grayscale compatibility. Specifically, Figure 3 has been significantly redesigned for clarity, and all figure captions have been reviewed to ensure they are self-explanatory.**

**Comment #7: The manuscript is verbose. Shorten methodological descriptions by moving details to supplementary materials.**

**Response: We sincerely thank the reviewer for this invaluable suggestion. We have substantially streamlined the entire manuscript by strategically moving technical details to the supplementary materials. Specifically:**

- **Detailed Parameter Calibration protocols (formerly Section 1.4) are now summarized.**
- **The detailed Ablation Study (formerly Section 1.6) is now summarized, with full details and literature comparison in Supporting Information S5.**
- **Specific Algorithmic Implementation details (formerly in Sections 2.2 & 2.3) are now summarized.**
- **Conclusion sections have also been carefully edited for conciseness.**

**We are confident these comprehensive revisions have made the manuscript more focused, readable, and impactful, while maintaining full scientific rigor through the detailed supplementary materials. We once again thank the reviewers for their guidance.**

**Sincerely,**

**Yuhong Chen (on behalf of all authors)
School of Economics and Management
Inner Mongolia University of Technology**
